# A bacterial artificial chromosome library for the Australian saltwater crocodile (*Crocodylus porosus*) and its utilization in gene isolation and genome characterization

**DOI:** 10.1186/1471-2164-10-S2-S9

**Published:** 2009-07-14

**Authors:** Xueyan Shan, David A Ray, John A Bunge, Daniel G Peterson

**Affiliations:** 1 Mississippi Genome Exploration Laboratory (MGEL), Department of Plant and Soil Sciences, Mississippi State University, Mississippi State, MS, USA; 2 Department of Biology, West Virginia University, Morgantown, WV, USA; 3 Department of Statistical Science, Cornell University, Ithaca, NY, USA; 4 Life Sciences & Biotechnology Institute; Institute for Digital Biology, Mississippi State University, Mississippi State, MS, USA

## Abstract

**Background:**

Crocodilians (Order Crocodylia) are an ancient vertebrate group of tremendous ecological, social, and evolutionary importance. They are the only extant reptilian members of Archosauria, a monophyletic group that also includes birds, dinosaurs, and pterosaurs. Consequently, crocodilian genomes represent a gateway through which the molecular evolution of avian lineages can be explored. To facilitate comparative genomics within Crocodylia and between crocodilians and other archosaurs, we have constructed a bacterial artificial chromosome (BAC) library for the Australian saltwater crocodile, *Crocodylus porosus*. This is the first BAC library for a crocodile and only the second BAC resource for a crocodilian.

**Results:**

The *C. porosus *BAC library consists of 101,760 individually archived clones stored in 384-well microtiter plates. *Not*I digestion of random clones indicates an average insert size of 102 kb. Based on a genome size estimate of 2778 Mb, the library affords 3.7 fold (3.7×) coverage of the *C. porosus *genome. To investigate the utility of the library in studying sequence distribution, probes derived from CR1a and CR1b, two crocodilian CR1-like retrotransposon subfamilies, were hybridized to *C. porosus *macroarrays. The results indicate that there are a minimum of 20,000 CR1a/b elements in *C. porosus *and that their distribution throughout the genome is decidedly non-random. To demonstrate the utility of the library in gene isolation, we probed the *C. porosus *macroarrays with an overgo designed from a *C-mos *(oocyte maturation factor) partial cDNA. A BAC containing *C-mos *was identified and the *C-mos *locus was sequenced. Nucleotide and amino acid sequence alignment of the *C. porosus C-mos *coding sequence with avian and reptilian *C-mos *orthologs reveals greater sequence similarity between *C. porosus *and birds (specifically chicken and zebra finch) than between *C. porosus *and squamates (green anole).

**Conclusion:**

We have demonstrated the utility of the *Crocodylus porosus *BAC library as a tool in genomics research. The BAC library should expedite complete genome sequencing of *C. porosus *and facilitate detailed analysis of genome evolution within Crocodylia and between crocodilians and diverse amniote lineages including birds, mammals, and other non-avian reptiles.

## Background

Crocodilians (Order Crocodylia) are a group of reptiles that originated roughly 200 million years ago [1,2]. They are apex predators in the marine and freshwater habitats in which they reside, and they play a major role in warm-water ecosystems throughout the world. There are 23 extant species grouped into three families – Crocodilidae (crocodiles), Alligatoridae (alligators and caimans), and Gavialidae (gharials) [3,4]. As evidenced by their frequent appearances in video documentaries and television programs, crocodilians are a subject of considerable human curiosity. Moreover, these reptiles have been common subjects/characters in mythology, folk tales, art (including cave paintings and hieroglyphics), and literature suggesting that they have considerable symbolic and practical significance in the lives of humans, past and present.

**Figure 1:**
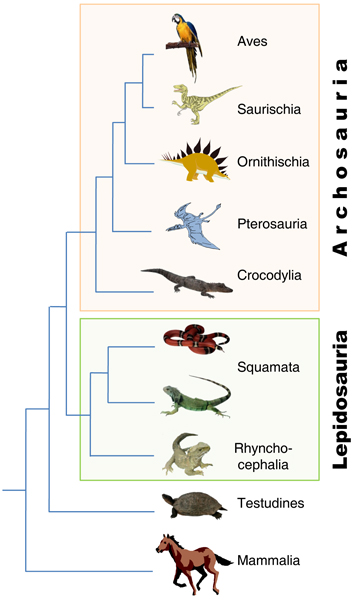
**A tree depicting potential relationships among amniotes**. Some relationships, especially the placement of Testudines [6,36], are controversial.

Crocodilians, birds, dinosaurs, and pterosaurs form a monophyletic group known as the Archosauria [5] of which only the crocodilian and the avian lineages are extant (Figure 1). In support of this premise, molecular phylogenetic evidence from nuclear and mitochondrial DNA sequencing indicates that crocodilians and birds are indeed each other's closest living relatives [5,6]. Among archosaurs, only the chicken and zebra finch have been the focus of complete genome sequencing efforts [7,8]. Genome level analyses of a crocodilian would be especially useful in leveraging information from the chicken, and a crocodilian genome would be the best possible outgroup for all genomic work within birds. However, with the modest exception of *Alligator mississippiensis *for which there is roughly 2.5 Mb of BAC end sequence [9] and 26 partially assembled BACs (GenBank, 10/3/2008), little sequence data is available for crocodilian genomes, in part due to a relative lack of high-quality molecular tools for this important clade.

For roughly 15 years, bacterial artificial chromosome (BAC) libraries have been the principal molecular substrate used in physical mapping and complete eukaryote genome sequencing [10]. Gridding of ordered BAC libraries (i.e., libraries in which each clone is stored in its own microtiter well) onto macroarrays and multiplex screening techniques have facilitated rapid gene isolation. The utility of BAC clones as substrates for end sequencing, in conjunction with advanced DNA fingerprinting techniques and macroarray analysis, has permitted construction of robust physical maps and selection of minimum tiling paths (*i.e*., sets of minimally overlapping BAC clones spanning entire chromosomes or chromosomal regions) for accurate genome sequencing and assembly. Recent advances in sequencing technologies (e.g., 454 pyrosequencing, Illumina sequencing, etc.) have created powerful opportunities in which ordered BAC libraries play a critical role. A particularly promising strategy for simultaneous physical mapping and sequencing of large eukaryotic genomes involves sequencing pools of sheared, individually "bar coded" BAC clones. After sequencing, those reads sharing a bar code (i.e., corresponding to the same BAC) are grouped together and assembled *in silico*, and physical maps are constructed by identifying overlapping assembled or partially assembled BAC sequences [11].

To expedite genome research in crocodilians, we have constructed a BAC library for the Australian saltwater crocodile (*Crocodylus porosus*). The *C. porosus *library is only the second large-insert DNA library for a crocodilian – a 10× library exists for *Alligator mississippiensis *[12] – and the only BAC library for Crocodilidae, the largest of the crocodilian families. *C. porosus *is the largest living crocodilian and, along with *A. mississippiensis*, the only crocodilian species to be commercially farmed. Here we describe generation and characterization of the *C. porosus *BAC library and demonstrate its utility as a tool for gene isolation, genome characterization, and comparative genomics.

## Methods

### Preparation of nuclei agarose plugs from crocodile blood sample

Whole blood was obtained from *Errol*, a male *C. porosus *from the Darwin Crocodile Farm [13] near Darwin, Australia. Blood was suspended in citrate buffer (250 mM sucrose, 40 mM trisodium citrate, pH 7.6) containing 5% v/v dimethylsulfoxide, aliquoted into 1.5 ml polypropylene tubes, flash frozen in liquid nitrogen, and shipped to the Mississippi Genome Exploration Laboratory [14]. One of the tubes was thawed on ice and centrifuged at 4,000 rpm in a microcentrifuge for 4 min. The supernatant was decanted, the pellet was gently re-suspended in 1 ml of STEX buffer (100 mM NaCl, 100 mM Tris-HCl, 100 mM EDTA, pH 8.0), and the mixture was centrifuged as described above. The blood cell pellet was re-suspended in 500 μl STEX buffer and placed in a water bath at 45°C. After 15 min, the blood suspension was mixed with an equal volume of 45°C 2% w/v Cambrex (Rockland, ME) SeaPlaque Agarose (cat. no. 50100) in STEX buffer. The mixture was poured into a small Petri dish so that the depth of the solution was roughly 2 mm. After 20 min at 4°C, the resulting gel was cut into 10 × 5 mm rectangles, and these "plugs" were transferred into a conical 50 ml polypropylene tube containing 40 ml of lysis buffer (STEX buffer containing 1% w/vN-lauroylsarcosine and 300 mg/ml proteinase K). The capped tube was incubated at 37°C overnight with gentle agitation. Plugs were transferred into 0.5 M EDTA (pH 8.0) containing 0.1 M phenylmethylsulfonyl fluoride and incubated at 4°C for one hour. Plugs were washed in 0.5 MEDTA (pH 8.0) and then stored at 4°C in this buffer.

### Preparation of high-molecular-weight insert DNA

A few test DNA plugs were exposed to different *Hind*III concentrations to determine conditions providing the largest number of fragments between 100 to 500 kb [see [15]]. The optimal enzyme concentration as determined in the test digests was used in a large-scale partial digest. Plugs used in the mass digestion were macerated and placed in a slot well of a 1% w/v Cambrex SeaKem Gold Agarose (cat. no. 50150) gel in 0.25 × TBE buffer (22.5 mM Tris, 22.5 mM boric acid, 0.5 mM EDTA, pH 8.0). Size selection of partially digested DNA was performed using pulsed-field gel electrophoresis (PFGE) according to Chalhoub et al. [16]. Size-selected *Hind*III fragments between 100 and 500 kb were recovered from agarose by electroelution according to Peterson et al. [15].

### BAC library construction

BAC library construction was performed as described in Peterson et al. [15] using the pIndigoBAC-5 vector (Epicentre, Madison WI) and ElectroMAX DH10B T1 Phage-Resistant Competent Cells (Invitrogen, Carlsbad, CA). Clone picking and library replication were performed using a Genetix QPixII robot (New Milton, Hampshire, UK). To monitor the quality of the BAC library and determine mean insert size, 96 BAC clones from every fiftieth 384-well plate were evaluated by *Not*I digestion and PFGE. For these analyses BAC DNA was isolated using an AutoGenprep 960 robot (AutoGen, Holliston, MA).

### Macroarray construction

High density macroarrays were prepared using a Genetix QPixII robot. Each array consisted of 18,432 double-spotted BAC clones stamped onto a 22.5 cm^2 ^Hybond N^+ ^filter (GE Healthcare, Piscataway, NJ). There were enough *C. porosus *BAC clones to produce five complete macroarrays (101,760 clones ÷ 18,432 clones/macroarray = 5.52). Stamped arrays were placed clone-side up on LB (Luria-Bertani) agar containing 12.5 mg/L chloramphenicol and incubated at 37°C overnight. Each macroarray was fixed via incubation in 0.5 N NaOH, 1.5 M NaCl for 7 min followed by incubation in 1.5 M NaCl, 0.5 M Tris Cl for 7 min. The membranes were allowed to air dry for 1 h, treated with 0.4 N NaOH for 20 min, and washed in 5× SSPE (0.75 M NaCl, 50 mM Na_2_HPO_4_, 5 mM EDTA, pH 7.4) for 7 min. Macroarrays were air dried and stored in sealed plastic bags.

### Probe design and BAC library screening

Overgo probes [17] were designed from the consensus sequences of CR1a and CR1b (see Additional File 1, Table S1), two recently active CR1 subfamilies present in the genomes of *Crocodylus moreletii *and *Osteolaemus tetraspis *(D. Ray, unpublished data), and from a partial cDNA sequence of the *C. porosus C-mos *gene [GenBank:AF478196]. The overgos (Table 1) were labeled with ^32^P and hybridized to macroarrays as previously described [18,19]. Probe hybridization images were recorded and analyzed using a Storm 820 (GE Healthcare, Piscataway, NJ) phosphoimager. For the CR1-probed macroarrays, the spot densitometry tool in AlphaEaseFC, Version 3.3.0 (Alpha Innotech, San Leandro, CA) was used to explore variation in spot (clone hybridization) intensity and estimate copy number as described previously [20]. Positive *C-mos *clones were confirmed by PCR using the CMF1/CMR1 primer pair (Table 1). PCR was performed using the following thermocycler series: 95°C denaturation for 5 min, 34 full PCR cycles (94°C for 1 min, 50°C for 1 min, and 72°C for 1.5 min), and a final 72°C 10 min extension step.

**Table 1:** Overgo and primer sequences

Name	Description	Sequence
CMOF	C-mos overgo, forward	TGGAGGATGGCTTATCTCTGAG
CMOR	C-mos overgo, reverse	GGCAAATATTGGGGCTCAGAGA
CR1aOF	CR1a overgo, forward	AGCGGAGGTGGTTCAAGCACCT
CR1aOR	CR1a overgo, reverse	AAGGTGTTCAATGTAGGTGCTT
CR1bOF	CR1b overgo, forward	AATAGGTCCAAGGAGGTGATAC
CR1bOR	CR1b overgo, reverse	CCGATAGAGGGGAAGTATCACC
CMF1	C-mos forward primer1	ATCACGGCAGAGCTTCTGGG
CMR1	C-mos reverse primer1	TGGCAAATATTGGGGCTCAG
CMF2	C-mos forward primer 2	GTTGTGCAAGATCGGAGACT
CMR2	C-mos reverse primer 2	GACGTAACTGGGCTACATTC

### Subcloning and sequencing of the *C. porosus C-mos *gene

A BAC clone containing the *C. porosus C-mos *gene was digested with *Bam*HI and *Hind*III at 37°C for 1 hr followed by heating to 65°C for 10 min. The cloning vector pCRII-TOPO (Invitrogen, Carlsbad, CA) was likewise double-digested, purified by electrophoresis on a 1% w/v agarose gel, and isolated from agarose using a Qiagen (Valencia, CA) QiaQuick Gel Extraction kit. Ligation was performed at 16°C for 16 hrs. The ligation mixture was used to transform chemically competent Invitrogen (Carlsbad, CA) One Shot TOP10 cells according to the manufacturer's instructions. Subclones were plated and 40 were screened by PCR using the *C-mos *primers CMF1 and CMR1 (Table 1). One positive subclone, which was shown by gel electrophoresis to contain a 3.7 kb insert, was sent to SeqWright (Houston, TX) for cycle sequencing using the CMF1 and CMR1 primers and two additional primers (CMF2 and CMR2 – Table 1). Use of a combination of primers was intended to extend the target area so that it would encompass the entire *C-mos *coding sequence and several hundred bases 5' and 3' of the coding region. Base-calling and assembly of sequence reads were performed using Phred and Phrap, respectively [21-23]. Trimming of the assembled *C-mos *sequence was conducted using Cross_Match [23]. The 3576 bp product was submitted to GenBank and assigned the accession FJ011695.

## Results and discussion

### BAC library coverage

**Figure 2:**
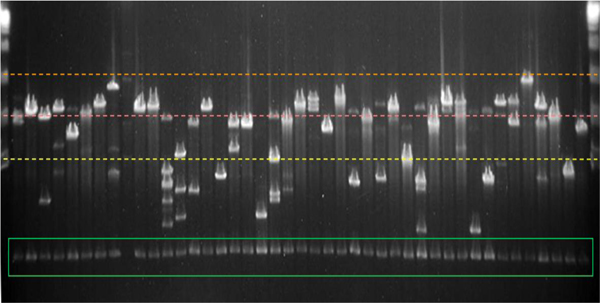
***Not*I digest of random *C. porosus *BAC clones**. The first and last lanes contain a DNA ladder. Molecular weights of 50, 100, and 150 kb are indicated by yellow, pink, and orange dotted lines, respectively. The 7.4 kb vector band is visible at the bottom of most lanes (green rectangle). The average insert size is 102 kb.

The *C. porosus *BAC library consists of 101,760 individual clones stored in 265 bar-coded 384-well microtiter plates. *Not*I digestion and PFGE of 768 BAC clones indicates an average clone insert size of 102 kb (Figure 2) with 70.5% of clones possessing inserts > 100 kb, 24.2% with inserts between 50 and 100 kb, and 5.3% with inserts smaller than 50 kb. Internal *Not*I sites are present in 41.6% of PFGE-examined BAC clones (Figure 2), and 1.3% of clones are probable false positives, i.e., they possess a vector band but no insert band(s). Collectively, the entire BAC library represents 10.2 Gb of crocodile genomic DNA [i.e., 101,760 clones•102 kb/clone•(1–0.013) = 10.2 Gb].

The exact genome size of *C. porosus *is unknown. However, measurements made for two other *Crocodylus *species (*C. siamensis *and *C. niloticus*) [24] are both 2778 Mb. Assuming the *C. porosus *genome size is similar to those of these closely allied taxa, we estimate that the library affords 3.7× coverage (i.e., 10.2 Gb ÷ 2778 Mb = 3.7) of the *C. porosus *genome. Theoretically, this level of coverage affords 98% probability of finding any given genomic sequence at least once in the library [25].

### Survey of CR1 elements in crocodile genome

CR1 elements are non-LTR retrotransposons existing in high copy numbers in bird and reptile genomes [26-28]; there are about 100,000 CR1 elements in the chicken genome [26]. CR1 retrotransposons are considered excellent markers for molecular phylogenetic and population genetic studies [29,30]. Initial studies on the sequences from the 21 BAC clones of *Alligator mississippiensis *available in GenBank [AC164519.3, AC154087.3, AC161341.3, AC165215.2, AC162159.2, AC155801.3, AC155802.2, AC154170.2, AC155800.2, AC155799.2, AC154169.2, AC154945.2, AC154088.2, AC149028.2, AC148923.3, AC149025.3, AC148578.2, AC149029.2, AC149026.2, AC148964.2, and AC149027.1] revealed that at least two CR1 subfamilies, referred to here as CR1a and CR1b, have recently been active in crocodilian genomes. This observation is consistent with Shedlock et al. (2007) in which the authors suggested that multiple CR1 lineages may have been active in alligators. In addition to *A. mississippiensis*, CR1a and CR1b have been identified in *Crocodylus moreletii*, and *Osteolaemus tetraspis *(D. Ray, unpublished) and consensus sequences for conserved regions of these elements have been generated (see Additional File 1, Table S1).

**Figure 3:**
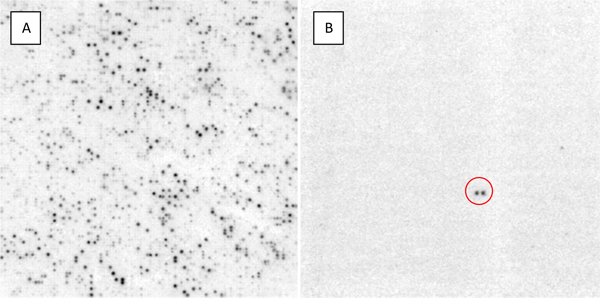
**Exploring the *C. porosus *genome using macroarray analysis**. (A) One-quarter of a *C. porosus *macroarray hybridized with the overgo probes CR1a and CR1b. (B) One-quarter of a macroarray showing a BAC (double-spot, red circle) recognized by our *C-mos *overgo. This BAC was isolated and used to sequence the complete *C-mos *gene.

**Figure 4:**
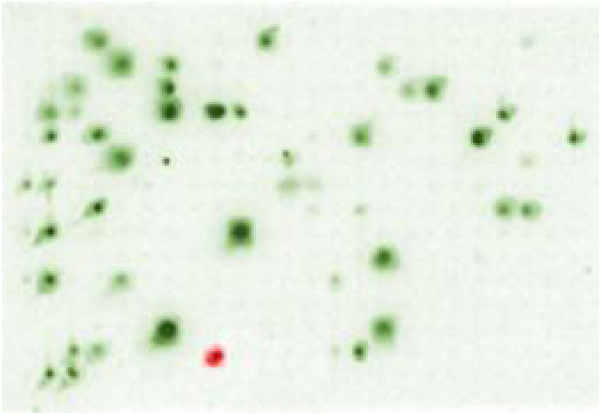
**Hybridization of CR1 and *C-mos *overgos to arrays of one 384-well microtiter plate**. Images were colorized and digitally merged using Adobe Photoshop. The BAC positive for *C-mos *is indicated in red while clones showing hybridization to CR1b are green. The *C-mos *clone exhibits no visible co-hybridization with CR1b.

To explore CR1 distribution in *C. porosus*, macroarrays were screened with CR1b-derived overgos or a combination of CR1a and CR1b overgos. Comparison of the CR1b- and CR1a/b-probed macroarrays revealed that the CR1a and CR1b subfamilies are not distinguishable in our assay, i.e., virtually no differences in hybridization pattern and intensity were observed when comparing the CR1b and CR1a/CR1b filters (data not shown). It was clear, however, that elements similar to CR1a and b are fairly abundant in *C. porosus*. Examination of one-quarter of the CR1a/b-probed macroarray (Figure 3A) indicates that 8.9% of clones show hybridization to the CR1a/b overgos while CR1b overgo hybridization to a filter stamped with the contents of a single 384-well plate from the BAC library suggests that 12.8% of clones (49 of 384) are positive for the CR1b overgo (Figure 4). Densitometric analysis of the macroarray reveals that there is a six-fold variation in positive clone hybridization intensity. If the lightest, but clearly positive hybridization signals represent clones containing one copy of a CR1a/b element and if the darkest clones contain six elements, there would be approximately 19,754 copies of CR1a/b in the *C. porosus *genome [see [20] for calculation method]. However, there is the possibility that macroarray exposure conditions were such that only clones with multiple copies of CR1a/b elements appear positive; inclusion of a single-copy sequence control on the macroarray could be used to test this possibility. Moreover, the use of overgos (each pair representing only 36 bp) rather than whole CR1 sequences clearly limits hybridization to those elements where the regions corresponding to the overgos have been conserved. Based on analysis of 2.5 Mb of BAC end sequence, Shedlock [9] estimated the number of CR1 elements in *Alligator mississippiensis *at 408,000 copies. While *A. mississippiensis *and *C. porosus *are separated by 100 million years of evolution [31], it seems reasonable that they might have similar total numbers of CR1 elements. Consequently, the *C. porosus *copy number estimate of 19,754 should be viewed as a minimum number of CR1a/b elements, not total CR1 elements, in the *C. porosus *genome.

Statistical analysis of the macroarray data indicates that CR1a/b elements are not randomly distributed throughout the *C. porosus *genome. The macroarray contains 18,432 individual clones with an average insert size of 102 kb. Consequently, a single macroarray represents roughly 0.68 genome equivalents, i.e., (18,432•102 kb) ÷ 2778 Mb. If the crocodile genome contains 19,754 copies of CR1a/b, then we would expect approximately 13,369 copies of CR1a/b per macroarray, and if these were distributed randomly we would expect, on average, 0.73 copies of CR1a/b per clone. However, only 8.9% of clones on the macroarray show hybridization to the CR1a/b probes. To test whether such a distribution is likely by chance, we can formulate the problem as a statistical "urn model." Suppose we have 18,432 urns, and we drop 13,369 balls into them at random. In such a case, classical statistical asymptotic theory [32] describes the distribution of the number of occupied (or empty) urns. In this experiment we found 1,203 occupied and 17,229 empty urns. The null and alternative hypotheses are as follows:

*H*_0_: The allocation is completely random;

*H*_A_: Not H_0_.

Under *H*_0_, the number of empty urns is (approximately) normally distributed with mean 8,924 and standard deviation ≈ 40 [33] (verified both theoretically and by simulation), so the observed number of empty urns (i.e., 17,229) is more than 200 standard deviations above the mean, and hence is almost impossible under *H*_0 _(the formal *p*-value is zero to 32 decimal places). We therefore conclude that the distribution of CR1a/b is non-random.

A project is underway that will involve sequencing some CR1 positive BAC clones from *C.porosus *so that we may (among other things) compare the structure and distribution of CR1 elements in the *Alligator *and *Crocodylus *genomes. The sequence data can then be used for evolutionary analyses among crocodilians, birds, and non-archosaur reptiles.

### Identification of a crocodile *C-mos *gene containing BAC clone

A randomly selected macroarray was hybridized with the *C-mos *overgos (Table 1), and fortuitously one of the 18,432 double-spotted clones on the array exhibited a positive signal (Figure 3). The plate and well address of the clone was determined based upon the macroarray number, the location of the positive signal on the macroarray, and the spatial relationship between the two spots [see [34]]. To make sure that the correct clone was identified, a hand-held plate gridding/replicating device was used to stamp two nylon filters with the clones in the 384-well plate believed to contain the clone of interest. Blot hybridization using the *C-mos *overgo probes verified that the plate and well address obtained from the filter were correct (Figure 4). The duplicate filter was probed with the CR1b overgos. Of note, the *C-mos *positive clone shows no visible hybridization with the CR1b sequence (Figure 4). PCR with the CMF1 and CMR1 primers was used to independently verify the presence of the *C-mos *locus in the positive BAC clone.

### The *C. porosus C-mos *gene

Subcloning was used to isolate a 3.7 kb region containing the *C-mos *locus. Cycle sequencing using the CMF1, CMF2, CMR1, and CMR2 primers (Table 1) and assembly of the reads resulted in production of a continuous 3,576 bp sequence [GenBank:FJ011695]. This sequence contains the entire coding sequence (CDS) for *C-mos *as well as > 1000 bp upstream and downstream of the CDS. Like most characterized *C-mos *sequences [35], *C. porosus C-mos *possesses a single ORF. The ORF, which starts at base 1313 and ends with the stop codon TGA (bases 2366–2368), codes for a 351 amino acid protein. Alignment of the *C. porosus C-mos *CDS with those of chicken, zebra finch, and green anole orthologs (see Additional File 1, Figures S1 and S2 for alignments) reveals considerable nucleotide and amino acid conservation (Table 2). Interestingly, the *C. porosus *CDS is six nucleotides (2 amino acids) longer than the chicken, zebra finch, and green anole *C-mos *genes. These two additional amino acids are adjacent to each other and are located at the C-terminal end of the *C. porosus C-mos *protein (see Additional File 1, Figures S1 and S2).

**Table 2:** Similarity of complete coding sequences of four *C-mos *genes

Organism	GenBank accession or reference to database from which sequence was mined	Nucleotide identities (%) with respect to *C. porosus*	Amino acid identities (%) with respect to *C. porosus*
*Crocodylus porosus*	FJ011695	100.0	100.0
*Gallus gallus*	M19412.1	77.9	76.5
*Taeniopygia guttata*	[37]	76.8	75.6
*Anolis carolinensis*	[37]	69.6	71.6

### Crocodilian sequence and comparative genomics

Comparative genomics research is a burgeoning field with high potential to increase our understanding of the structure, function, and evolution behind the diversity of life. However, the primary focus of most efforts over the past several years has been on comparisons among mammals. For example, Miller et al. recently created a 28-way alignment of available vertebrate genomes in which only eight taxa, *Gallus*, *Anolis*, *Xenopus *and five fish represent the entirety of non-mammalian vertebrates. Understanding the evolution and interrelationships among all amnotes will be severely hindered by this lack of diversity. Ongoing projects to sequence the green anole (Sqamata) and the painted turtle (Chelonia) will help correct the disparity but one significant lineage of the amniote tree remains to be addressed – Crocodylia. It is our hope that the generation of this library will facilitate genomics research in this critical lineage.

## Conclusion

We have constructed a high quality 3.7× BAC library for *Crocodylus porosus *and demonstrated the library's utility as a genomics tool. We are currently screening the library with other genes and repeat sequences as a means of investigating the structure of the Australian saltwater crocodile genome and facilitating comparative genomics research among archosaurs. Copies of the BAC library, individual clones, and macroarrays can be obtained from the Mississippi Genome Exploration Laboratory [14].

## Competing interests

The authors declare that they have no competing interests.

## Authors' contributions

The project was conceived through discussions between DGP and DAR. XS constructed the BAC library and performed all bench research in the lab of DGP. Experiments were designed by XS with the input of DGP and DAR. DAR provided sequence data for CR1 analyses of the library and made arrangements associated with acquisition of crocodile blood. JAB performed the statistical analysis associated with CR1 distribution. All authors were involved in manuscript writing and editing. The final version of the manuscript was read and approved by all authors.

## Supplementary Material

Additional file 1 **Table S1**. Consensus sequences of highly conserved portions of crocodilian CR1a and CR1b subfamily elements. **Table S2**. Putative C-mos CDS for platypus mined from the current genome assembly amended so that the two overlapping but out of frame ORFs are combined into a single presumed C-mos CDS. **Figure S1**. MUSCLE-based nucleotide alignment of the complete coding sequences of *A. carolinensis*, *C. porosus*, *G. gallus*, and *T. guttata *C-mos genes. **Figure S2**. MUSCLE-based amino acid alignment of the complete coding sequences of *A. carolinensis*, *C. porosus*, *G. gallus*, and *T. guttata *C-mos genes. Click here for file
