# Supplementary material for: A bacterial artificial chromosome library for the Australian saltwater crocodile (Crocodylus porosus) and its utilization in gene isolation and genome characterization
Source: BMC Genomics. 2009 Jul 14;10(Suppl 2):S9. doi: 10.1186/1471-2164-10-S2-S9 (PMC2966330; doi:10.1186/1471-2164-10-S2-S9)
Supplement: Additional file 1 — Table S1. Consensus sequences of highly conserved portions of crocodilian CR1a and CR1b subfamily elements. Table S2. Putative C-mos CDS for platypus mined from the current genome assembly amended so that the two overlapping but out of frame ORFs are combined into a single presumed C-mos CDS. Figure S1. MUSCLE-based nucleotide alignment of the complete coding sequences of A. carolinensis, C. porosus, G. gallus, and T. guttata C-mos genes. Figure S2. MUSCLE-based amino acid alignment of the complete coding sequences of A. carolinensis, C. porosus, G. gallus, and T. guttata C-mos genes. [file 1471-2164-10-S2-S9-S1.pdf]

ADDITIONAL FILE 1

Shan X, Ray DA, Bunge JB, and Peterson DG

Table S1: CR1a and CR1b consensus sequences

```
>Croc_CR1a_Consensus
CTCAAGCAAATCCCGGGACGTCATTCTCCCCTTGTACTCGGCCTTGGTGAGGCCGCAGCTGGAGTACTGCGTCCAGTTTTGGGC
TCCACAATTCAAAAAGGATGTGGAGAAGCTTGAGAGAGTCCAGAGGAGAGCCACGCGCATGATCAGAGGTCAGGAAAACAG
ATCTTACGATGACAGGCTGAGAGCCATGGGACTCTTTAGCCTGGAAAAGCGCAGGCTCAGGGGTGATCTGATGGCCACCTAT
AAGTTTATCAGGGGTGACCACCAGGATCTGGGGGAACGTTTGTTCACCAGAGCGCCCCAAGGGATGACGAGGTCGAACGGTC
ACAAACTCCTGCAAGACCGTTTCAGGCTGGACATAAGGAAGAATTTCTTTACTGTCCGAGCCCCCAAGGTCTGGAACAGTCTG
CCAGCGGAGGTGGTTCAAGCACCTACATTGAACACCTTCAAGAGTAAATTGGATGCTTATCTTGCTGGGATCCTATGACCCCAG
CTGACTTCCTGCCCTTTGGGC

>Croc_CR1b_Consensus
GACGAATAGGTCCAAGGAGGTGATACTTCCCCTCTATCGGGCGCTGGTCAGACCGCAGCTGGAGTACTGCGTGCAATTCTGG
GCGCCGCACTTCACGAAGGATGCGGATAACCTGGAGAGGGTCCAGAGAAGGGCCACTCGTATGGTTAAGGGCCTGCAGGCC
AAGCCCTATGAGGAGAGACTAGAGAAACTGGACCTTTTCAGCCTCCGCAAGAGAAGGTTGAGAGGCGACCTGGTGGCTGCCT
ATAAGTTCATCAGGGGGGCACAGAAGGGAATTGGTGAGTATTTATTCACCAAGGCCCCCCGGGGGTTACAAGAAATAATGG
CCACAAGCTAGAAGAGAGCAGATTTAGATTGGATATTAGGAGGAACTTCTTCACAGTTCGAGTGGCCAAGGTCTGGAACGGG
CTCCCAAGGGAGGTGGTGCTCTCCCCTACCCTGGGGGTCTTCAAGCGAAGGTTAGATGTGTATCTAGCTGGGGTAATGTAGGC
CCAGCACTCTTTCCTGCCTATGCAGGGGG
```

Table S2: Putative platypus C-mos consensus sequence*

>Putative platypus C-mos sequence, range=chr7:6550872-6553730
TAATTTCCTATATATGCTGACTAGAATGTCACAGATTCTGTTCTCTTTCTGGTGTGTGTGTGTATCCTGACTGGAGTATACCAGT
GATTTTGTTTGGATCAATTATATTTAGGTTGCCATTTTTTACAAGCTGAATGTTACACCTAATTATTCCCTTAATAGGTTAAACAA
ATGTCTGCTGTCTGTGTTTCAGCTCTTTCTGCCAGTAACTGCTGGAAGTTATTTAATGCAGCTGGAGGCTTACATTGACAGTCAT
TATGGCCTGCAAAGTGTGAAATGTTTCATTTTACTGCTGTGATCCCTGTAGTCTAAGGACTGATAACATTCAACGGAAGAGATT
TGAAAGTGCATTTTACTATAATTAGAACAATGGTGTAATAGGCACATTATGAGGTGTACCGGGATACCCGGTTTATGCCCGAA
ACCTAAGAGCTTGTTTTTATAAAAATTCACTTTTAGTGTGTGAGGAAAATCTTTTCAGCTGCAAATGATTGGCCTTTCGTGTGGA
GATTATTCCCCCCACCCACCCAAGTCTTTCTGATAAACCTTCACACACACACACACACACACCCCCTCAAAGCAGCCACAGTTGT
AGCTCTTTTGGAGAAAATGAAGCAAGGAAATAAAGGAACAATGGAGGGCAAGCAGGCTGTAGTGTTTCAGGACTCGCTCAGC
TAGCTGGTGGGGGTAGGGAGGTGGGGAAGAGAATAGTGGCTCCACTTCCCGTGTGGGGCTCCATGCCTTCTCCCATCCCCCT
GACTCGCTTCCTTCCCCGAGAGTTTTCCCCCTCCTTGGACTCGCGGCCATGCAGCAGCCCCTTGGAGCGGCCTGGCCGAGCCGG
GGCCCTCTCCCTGGGTGGGACGCCTTTCCCCAGGGCCCGCCGGTTACCCCCGCGGCTGGCCTGGTGTTCCATCGACTGGGAAG
GGGTGTGCTTCCTTCAGAGGCTGGGGGCCGGGGGGTTTGGCTCCGTCTACAAGGCCACCTACCACGGGGTCACGGTGGCCGT
CAAGCAGGTGAAGAAGTGCAGCAAGAACCGGCTGGCGTCCAGGCAGAGCTTCTGGGCCGAGCTGAACGTGGCCCGGCTCCG
CCACGCCAACGTCGTGCGGGTCGTGGCTGCCAGCACGTGCGCCCCCGCTGACCATGACAGCCTGGGGACCATCATCATGGAG
TACACGGGCAGCACCACCCTGCATCACGTCATCTACGGGGCCAGCTCTTGCCAAGAGGAGGGCGGAGGCGGTGGGCAGAGG
GTGCTTAAACCTGGTCACCTGTCTGAAGTACTCTCTGGATGTCGCAAGCGGATTGCTCTTTCTTCACTCCCAAGGCGTCGTTCAC
TTGGACCTGAAGCCAGCCAACATCTTGATTACTGAGCGGGACACCTGCAAAATCGGTGACTTCGGGTGCTCTCAGAGACTAGA
AGATCCGGTGGGCTCCGGCCCCCAGCACTGCCACCTGGGAGGCACCTACACCCATCGAGCCCCCGAGCTCCTGAAGGGAGAG
ACGGTCACCCCCAAAGCAGACATTTACTCTTTCGCTATCACGCTCTGGCAGATGGCCACCAAGGAGGTACCTTATTCGGGGGA
CCGCCACTATGTGGTCTACGCCGTGGTGGCCTATAACCTGCGCCCTTCCCTCTCGGCTGCCATCTTCTCCGAATCTCCCCCCGGC
CAAAGGCTGCAGGGCATCATCGACCGCTGCTGGGCATCCAGCACCCTGCATCGACCCACCGCGGAACTCCTCCTCCGTGACCT
CGGCTCTTTGAGCGCTGATTTGGGCCGGCTCTACTCCTGAAACCTGCACCCAAAACAGATGAGCCCTCTTTGTGTTTTACCTGTC
TTGTTCTTGTTCCTGGTTTTCTCGTTCTTTCGGTTCCAGAAGATGGCCAGGTAGAGGGAGGAAAATATGCTGTGGAATGGAAGT
TAAACTCTTATAAAAAATAAAGTGACTTTAAAAGACAGTTTGTTAGCCCTAGTTGAATCGCTTGACTAGGAAAACCCTGGCCCA
GTTTTTAATCGGTAGTACTGGAGTGCTTACTCTGTGCAGAGCACTGTTCTAAGCACTAGGGATAGTACAAAAAAAGAAGATAA
GATCCCTGTGTCCTTGAGAAGCATGGTCTACTAACTCCCCTCAAAGCCTCTCTGAACCTGCTCCATTTTAACCGATCCACACTGT
TAAACCTATGCCAATGTGCAACCTTATAAATGAGGGATTTGCTCAAATGAACTTAGTTGAAATTAAAAGGAGTCCAAAGACTTC
AGAGCCATTTCTAGAAGCCTAGGCAATGCTTTATCTCACTACTAGGTTAACTGTTCCCAATTCCAAAGAATTTAAGCCAGCAGCT
GATGATCAAATTAGATATATCTATAAATACAATTATTTTAAAGCCCTTCTAAATCCTTTCTAATTCTTAAACCTCTGCTTATGATG
AGTTACTAAGAAGAACCCAGGATTGCAAAATTTGTGCCTGCCTTATCACACTCCCTCAGTACTGAAAAATTAAATCAAGCGACT
GTATTCCTGCTAATACTAAGCTGGCATCTAGCTATTTTGGTTTTCAGGGTTAAAAAAAAATGGGAAAATGAGCAACTTTGAAGT
TTGAAATAATGAGGGCTTGAAGCTCTGAGAAGCT

*Note that this sequence differs from that in the current (8/17/08) platypus assembly (see Materials and Methods for explanation).

Figure S1. MUSCLE nucleotide alignment of the complete coding regions of the Anolis carolinensis, Crocodylus porosus, Gallus gallus, and Taeniopygia guttata C-mos genes.

```
                10        20        30        40        50        60        70
  1  ATGCCATCCCCTATCCCCTTTAAACGTTTTTTGCCCCCAGAGTTTTCCCCTTCTGTGGACTCACGGCCCT  Crocodylus
  1  ATGCCATCTCCTCTTCCTCATAATCGTCTTCTTCATCTACAATTTTCTTCATCTGTGAATGCACGGCCCT  Anolis
  1  ATGCCATCACCTATTCCTTTTAATAGCTTTCTTCCTTTGGAGCTCTCCCCATCTGCAGACTTGAGACCTT  Gallus
  1  ATGCCATCACCTATTCCACTTAATTGTTTTCTTTCTTTTGAGTATTCCCCCTCTGCGAATTTGCGCCCCT  Taeniopyg.

                80        90       100       110       120       130       140
 71  GCAGCAGCCCCTTGGCACTTCCTGGCAAAGGTGGTAAACACTTCCTGGGAGAAATCCCTTCCCCCAGGGC  Crocodylus
 71  GCAGTAGTCCCTTGTTGTTTCCAGACAAAGCTGAAAAGTTTTTTTGCGCCGAAGGTGTTTCGCCCAGGGT  Anolis
 71  GCAGCAGCCCTGTAGTTATCCCTGGCAAAGATGGAAAAGCCTTCCTAGGAGGGACCCCTTCACCCAGGAC  Gallus
 71  GCAGCAGCCCTTTAATTATCCCTGGCAAAGACAGCAAAAGCTTTCTGGGGGGAGCTTCTTCAGCCAGGAC  Taeniopyg.

               150       160       170       180       190       200       210
141  TCGCCGGCTGCCCCCCCGCCTGGCTTGGTGCTCCATAGATTGGGATCAAGTCTGCCTACTGCAGCCCTTG  Crocodylus
141  TCGCCGACTACCGACTCGCCTAGCTTGGTGTTCAATAGACTGGGATCAGTTGTGTTTACTACACCCCCTG  Anolis
141  ACGCCGTCTGCCCCCACGCTTGGCCTGGTGCTCCATCGACTGGGATCGGCTCTGCCTCCTGCAGCCCCTG  Gallus
141  TCGTCGCTTGCCCTCACGCTTGTCCTGGTGCTCCATTGATTGGGAGCAGCTCTGCCTCCTGCAGCCCTTA  Taeniopyg.

               220       230       240       250       260       270       280
211  GGCTCTGGGGGCTTTGGTTCGGTCTATAAGGCTACTTATCATGGGGAGATAGTTGCTGTGAAGCAGGTAA  Crocodylus
211  GGATCAGGTGGCTTTGGTTCAGTCTACAAGGCTATTTATCATGGAACTACAGTGGCAGTAAAGCAGGTGA  Anolis
211  GGCTCTGGGGGCTTTGGGGCTGTCTACAAGGCCACCTACCACGGTGTGACTGTGGCTGTGAAGCAGGTGA  Gallus
211  GGATCTGGGGGCTTTGGCTCTGTCTACAAAGCCACCTACCATGGTGCAACGGTGGCTGTGAAGCAGGTGA  Taeniopyg.

               290       300       310       320       330       340       350
281  AGAAGTGCAGCAAGAACCGTCTGGCATCACGGCAGAGCTTCTGGGCTGAGCTGAATGTAGCCCAGTTACG  Crocodylus
281  AGAAAAGCAGTAAGAACAGTTTGGCATCACGGCAGAGTTTCTGGGCAGAATTAAATGTGGCACATCTTGA  Anolis
281  AGAAGAGCAGCAAAAACCGGCTGGCATCCCGACAGAGCTTCTGGGCTGAGCTGAACGTAGCCCGACTGCA  Gallus
281  AGAAGAGCAGCAAAAACCGGCTGGCATCGCGGCAGAGCTTCTGGGCTGAGCTGAACGTGGCCCAGCTCCA  Taeniopyg.

               360       370       380       390       400       410       420
351  TCACAAAAACGTGGTACGTGTTGTGGCTGCCAGCACGTGTGCCCCGGCTGGCCAGGACAGCTTGGGTACT  Crocodylus
351  CCATAATAATGTGGTACGTTTAGTAGCTGCTAGCACATGTGCCCCTGCCAGTCAGGACACTTTGGGCACC  Anolis
351  GCATGATAATGTGGTGCGTGTGGTGGCTGCTAGCACGTGTGCCCCTGCCAGCCAGAACAGCCTGGGCACC  Gallus
351  ACACGATAATGTGGTACGTGTGGTGGCTGCCAGCACCTGTGCCCCAGCCAGCGAGAACAGCCTGGGCACC  Taeniopyg.

               430       440       450       460       470       480       490
421  ATCATCATGGAATATGCAGGCAGTGTTACCCTGCACCATGTCATCTATGGTACTGGCTGGGCACAGAGAA  Crocodylus
421  ATAATAATGGAATATGTAGGTAACAGTACTCTGCACCATGTTATCTATGGGACTGACTGCAAGACAGAAA  Anolis
421  ATCATCATGGAGTATGTTGGCAATGTCACCCTGCACCATGTCATCTACGGCACTAGAGATGCGTGGAGGC  Gallus
421  ATCATCATGGAGTACGTGGGTAACATCACTCTGCACCATGTAATTTATGGCACTGGGGGTGCATGGAGAC  Taeniopyg.
```

```
                500       510       520       530       540       550       560
491  AGGAAGATGATGATGGGGGAGGATGCAGCCAAGAGGTCCTGAGCATGCCTGAGTCCCTACGCTATTCCTG  Crocodylus
491  AAAGTAAGGACGATGGGCTTGGAAGTGGCCATGTATCTTTGAGCATAAGTGAGGCATTAGGCTATTCTTG  Anolis
491  AGGGCGAGGAGGAGGAAGGAGGATGTGGGAGGAAGGCTCTGAGCATGGCGGAGGCTGTGTGCTACTCGTG  Gallus
491  AGGGGGAGGATGATGAAGAAGGATGTGGAAGGAAGGCCCTGAGCATGGAACAGACTGTGTGCTACTCATG  Taeniopyg.
```

```
                570       580       590       600       610       620       630
561  TGACATTGTGACTGGCTTAGCCTTTCTTCACTCACAATGCATTGTGCACCTGGACCTGAAGCCTGCCAAT  Crocodylus
561  TGACATTGTAGCAGGTCTCATCTTCCTTCATTCGCAAACGATTGTGCATTTGGATTTAAAACCTGCTAAT  Anolis
561  TGACATCGTGACTGGCTTAGCCTTCCTTCACTCGCAGGGCATCGTGCACCTCGACCTGAAGCCTGCCAAT  Gallus
561  TGACATCATGACTGGCTTAGCCTTTCTGCACTCACAGGACATTGTGCACTTGGACCTGAAGCCTGCAAAT  Taeniopyg.
```

```
                640       650       660       670       680       690       700
631  GTGTTCATCACTGAGCAGGGGTTGTGCAAGATCGGAGACTTCGGGTGCTCCCAGAAACTGGAGGATGGCT  Crocodylus
631  GTATTCATCACTGAACAAAATATTTGCAAACTTGGAGACTTTGGATGCTCCCAAAAACTACAGGATCTTG  Anolis
631  ATCCTCATCACTGAGCACGGAGCGTGCAAGATCGGAGACTTCGGCTGCTCCCAGAGACTGGAGGAGGGCT  Gallus
631  GTTTTTATCACTGAGCAAGGAGTGTGCAAGATTGGAGACTTTGGGTGCTCCCAGAAACTGGAGATGGGCT  Taeniopyg.
```

```
                710       720       730       740       750       760       770
701  TATCTCTGAGCCCCAATATTTGCCAGCAAGGGGGCACGTATACACACCGTGCCCCAGAGCTCCTCAAGGG  Crocodylus
701  AATCATCAAGTCCACTACTTTCTCAACAAGGTGGGACATACACCCACCGTGCTCCTGAACTCCTTAAAGG  Anolis
701  TGTCCCAGAGCCACCATGTTTGCCAGCAAGGGGGCACGTACACCCACCGCGCTCCTGAGCTCCTCAAGGG  Gallus
701  CATCCCAGAGCGCCCGTGTTTGCCAGCAGGGGGGCACATACACACACCGTGCCCCTGAGCTCCTCAAAGG  Taeniopyg.
```

```
                780       790       800       810       820       830       840
771  GGAGAGGGTCACCCCCAAAGCAGACATTTACTCCTTTGCTATCACCCTCTGGCAAGTGGTAACACAAGAG  Crocodylus
771  TGAGAGAGTCACCCCTAAGGCTGACATTTACTCTTTTGCTATCACACTCTGGCAAATGGTTACGCAGCAA  Anolis
771  CGAGAGGGTCACTGCCAAAGCAGACATCTACTCCTTTGCCATCACCCTCTGGCAGATCGTCATGCGGGAG  Gallus
771  GGAGAGGGTCACTGCCAAAGCAGACATCTACTCATTTGCCATCACCCTCTGGCAGATGGTCACGCGGGAG  Taeniopyg.
```

```
                850       860       870       880       890       900       910
841  CAGCCCTATTTGGGGGAGCGCCAGTACGTGCTCTATGCTGTGGTGGCCTATAACTTGCGTCCCTCTCTGA  Crocodylus
841  GAACCTTATCTGGGTGAGCGCCAGTATGTACTCTACTCTGTGGTAGCTTGTAATCTGCGCCCTTCACTAA  Anolis
841  CAGCCCTACCTGGGCGAGCGGCAGTACGTGCTCTATGCTGTGGTAGCCTACAACTTGCGCCCTCCTCTGG  Gallus
841  CAGCCGTACCTGGGCGAGCGGCAGCACGTGCTCTACGCCGTGGTCGCCTACAACCTGCGCCCTTCGCTGG  Taeniopyg.
```

```
                920       930       940       950       960       970       980
911  GCGCTGCAGTCTTCTCTGGGTCACCCTCAGGCCGCAGACTTGAGAGCATAATTGGTAGCTGCTGGAGAGC  Crocodylus
911  CTGCAGATGTGTTTAAAGGTTCAGCCACTGGACAACAGCTTGAAACCTTAATTGAAAGTTGCTGGAAATC  Anolis
911  CGGCCGCCATCTTCCACGAGTCAGCGGTGGGCCAAAGGCTTCGGAGCATCATCAGCTGCTGCTGGAAGGC  Gallus
911  CTGCTGAGGAGTTCCACGGGTCACCAGCGGGCCAGACTCTGCACAGCATCATCAGCTGCTGCTGGAAGGC  Taeniopyg.
```

```
                 990       1000      1010      1020      1030      1040      1050
                   .         .         .         .         .         .         .
981   TGAGGCTGAGGAGCGCCCTACTGCAGCACAGCTGCTTCATAACCTCTCTTCCCTGCAGGATAGACTCTGG  Crocodylus
981   TGATGTTGCCAAACGTCCTAGTGCAGAACTTCTCCTCCAGAATATTCATTCTCTGTCTCTAAAAGTATAA  Anolis
981   TGACGTAGAGGAGCGCCTCAGCGCGGCCCAGCTGCTCCCCAGCCTCAGGGCCCTGAAGGAGAACCTCTAG  Gallus
981   CAATGCGGAGGAGCGCCTGCCTGCAGACCAGCTGCTTCCCAGCCTCAGGGCCCTCAAGCAGAGCCTCTAG  Taeniopyg.

1051  TACTGA  Crocodylus
1051          Anolis
1051          Gallus
1051          Taeniopyg.
```

X non conserved
X similar
X conserved
X all match

Figure S2. MUSCLE amino acid alignment of the complete coding regions of the Anolis carolinensis, Crocodylus porosus, Gallus gallus, and Taeniopygia guttata C-mos genes.

```
                    10        20        30        40        50        60        70
                     .         .         .         .         .         .         .
  1  MPSPIPFKRFLPPEFSPSVDSRPCSSPLALPGKGGKHFLGEIPSPRARRLPPRLAWCSIDWDQVCLLQPL  Crocodylus
  1  MPSPLPHNRLLHLQFSSSVNARPCSSPLLFPDKAEKFFCAEGVSPRVRRLPTRLAWCSIDWDQLCLLHPL  Anolis
  1  MPSPIPFNSFLPLELSPSADLRPCSSPVVIPGKDGKAFLGGTPSPRTRRLPPRLAWCSIDWDRLCLLQPL  Gallus
  1  MPSPIPLNCFLSFEYSPSANLRPCSSPLIIPGKDSKSFLGGASSARTRRLPSRLSWCSIDWEQLCLLQPL  Taeniopyg.

                    80        90       100       110       120       130       140
                     .         .         .         .         .         .         .
 71  GSGGFGSVYKATYHGEIVAVKQVKKCSKNRLASRQSFWAELNVAQLRHKNVVRVVAASTCAPAGQDSLGT  Crocodylus
 71  GSGGFGSVYKAIYHGTTVAVKQVKKSSKNSLASRQSFWAELNVAHLDHNNVVRLVAASTCAPASQDTLGT  Anolis
 71  GSGGFGAVYKATYHGVTVAVKQVKKSSKNRLASRQSFWAELNVARLQHDNVVRVVAASTCAPASQNSLGT  Gallus
 71  GSGGFGSVYKATYHGATVAVKQVKKSSKNRLASRQSFWAELNVAQLQHDNVVRVVAASTCAPASENSLGT  Taeniopyg.

                   150       160       170       180       190       200       210
                     .         .         .         .         .         .         .
141  IIMEYAGSVTLHHVIYGTGWAQRKEDDDGGGCSQEVLSMPESLRYSCDIVTGLAFLHSQCIVHLDLKPAN  Crocodylus
141  IIMEYVGNSTLHHVIYGTDCKTEKSKDDGLGSGHVSLSISEALGYSCDIVAGLIFLHSQTIVHLDLKPAN  Anolis
141  IIMEYVGNVTLHHVIYGTRDAWRQGEEEEGGCGRKALSMAEAVCYSCDIVTGLAFLHSQGIVHLDLKPAN  Gallus
141  IIMEYVGNITLHHVIYGTGGAWRQGEDDEEGCGRKALSMEQTVCYSCDIMTGLAFLHSQDIVHLDLKPAN  Taeniopyg.

                   220       230       240       250       260       270       280
                     .         .         .         .         .         .         .
211  VFITEQGLCKIGDFGCSQKLEDGLSLSPNICQQGGTYTHRAPELLKGERVTPKADIYSFAITLWQVVTQE  Crocodylus
211  VFITEQNICKLGDFGCSQKLQDLESSSPLLSQQGGTYTHRAPELLKGERVTPKADIYSFAITLWQMVTQQ  Anolis
211  ILITEHGACKIGDFGCSQRLEEGLSQSHHVCQQGGTYTHRAPELLKGERVTAKADIYSFAITLWQIVMRE  Gallus
211  VFITEQGVCKIGDFGCSQKLEMGSSQSARVCQQGGTYTHRAPELLKGERVTAKADIYSFAITLWQMVTRE  Taeniopyg.

                   290       300       310       320       330       340       350
                     .         .         .         .         .         .         .
281  QPYLGERQYVLYAVVAYNLRPSLSAAVFSGSPSGRRLESIIGSCWRAEAEERPTAAQLLHNLSSLQDRLW  Crocodylus
281  EPYLGERQYVLYSVVACNLRPSLTADVFKGSATGQQLETLIESCWKSDVAKRPSAELLLQNIHSLSLKV   Anolis
281  QPYLGERQYVLYAVVAYNLRPPLAAAIFHESAVGQRLRSIISCCWKADVEERLSAAQLLPSLRALKENL   Gallus
281  QPYLGERQHVLYAVVAYNLRPSLAAEEFHGSPAGQTLHSIISCCWKANAEERLPADQLLPSLRALKQSL   Taeniopyg.

351  Y  Crocodylus
350     Anolis
350     Gallus
350     Taeniopyg.
```

X non conserved
X similar
X conserved
X all match
